# Marital Histories and Associations With Later-Life Dementia and Mild Cognitive Impairment Risk in the HUNT4 70+ Study in Norway

**DOI:** 10.1177/08982643221131926

**Published:** 2022-11-02

**Authors:** Vegard Skirbekk, Catherine E. Bowen, Asta Håberg, Astanand Jugessur, Bo Engdahl, Bernt Bratsberg, Ekaterina Zotcheva, Geir Selbæk, Hans-Peter Kohler, Jordan Weiss, Jennifer R. Harris, Sarah E. Tom, Steinar Krokstad, Yaakov Stern, Bjørn Heine Strand

**Affiliations:** 1 25563 Division for Mental and Physical Health, Norwegian Institute of Public Health, Oslo, Norway; 2 Norwegian National Centre for Ageing and Health, 60512 Vestfold Hospital Trust, Tønsberg, Norway; 3 PROMENTA Research Center, Department of Psychology, University of Oslo, Oslo, Norway; 4 Independent Researcher, Vienna, Austria; 5 Department of Neuromedicine and Movement Science, Faculty of Medicine and Health Sciences, NTNU Norwegian University of Science and Technology, Trondheim, Norway; 6 Ragnar Frisch Center for Economic Research, Oslo, Norway; 7 Department of Geriatric Medicine, Oslo University Hospital, Oslo, Norway; 8 Faculty of Medicine, University of Oslo, Oslo, Norway; 9 Population Aging Research Center and Department of Sociology, University of Pennsylvania, Philadelphia, PA, USA; 10 Stanford Center on Longevity, Stanford University; 11 Department of Neurology, Columbia University Vagelos College of Physicians and Surgeons, USA; 12 HUNT Research Centre, Department of Public Health and Nursing, Faculty of Medicine and Health Sciences, NTNU Norwegian University of Science and Technology, Trondheim, Norway; 13 Levanger Hospital, Nord-Trøndelag Hospital Trust, Norway; 14 Department of Global Public Health and Primary Care, University of Bergen, Bergen, Norway

**Keywords:** mild cognitive impairment, dementia, marital status, population-based study, cohort study

## Abstract

**Objectives: **Earlier studies suggest that being married in later life protects against dementia, and that being single in old age increases the risk of dementia. In this study, we examine midlife marital status trajectories and their association with dementia and mild cognitive impairment (MCI) at ages 70 plus using a large population based sample from Norway. **Methods: **Based on a general population sample linked to population registries (*N* = 8706), we used multinomial logistic regression to examine the associations between six types of marital trajectories (unmarried, continuously divorced, intermittently divorced, widowed, continuously married, intermittently married) between age 44 and 68 years from national registries and a clinical dementia or a MCI diagnosis after age 70. We estimated relative risk ratios (RRR) and used mediation analyses adjusting for education, number of children, smoking, hypertension, obesity, physical inactivity, diabetes, mental distress, and having no close friends in midlife. Inverse probability weighting and multiple imputations were applied. The population attributable fraction was estimated to assess the potential reduction in dementia cases due to marital histories. **Results: **Overall, 11.6% of the participants were diagnosed with dementia and 35.3% with MCI. Dementia prevalence was lowest among the continuously married (11.2%). Adjusting for confounders, the risk of dementia was higher for the unmarried (RRR = 1.73; 95% CI: 1.24, 2.40), continuously divorced (RRR = 1.66; 95% CI: 1.14, 2.43), and intermittently divorced (RRR = 1.50; 95% CI: 1.09, 2.06) compared to the continuously married. In general, marital trajectory was less associated with MCI than with dementia. In the counterfactual scenario, where all participants had the same risk of receiving a dementia diagnosis as the continuously married group, there would be 6.0% fewer dementia cases. **Discussion: **Our data confirm that staying married in midlife is associated with a lower risk of dementia and that divorced people account for a substantial share of dementia cases.

## Introduction

Dementia is a progressive neurodegenerative syndrome affecting a large proportion of the older population. Global estimates suggest that 57.4 million people currently have dementia, and this number is projected to increase to 152.8 million in 2050 due to population aging (World Health Organization, 2021). In the absence of effective pharmacological treatments for dementia (Livingston et al., 2020), efforts to prevent or delay dementia (Lissek & Suchan, 2021) by reducing health and social risk factors have become a major research focus (Livingston et al., 2020). Mild cognitive impairment (MCI) is a cognitive diagnosis in older adults who do not fulfill the diagnostic criteria for dementia (Ganguli et al., 2011; Gates et al., 2019). MCI represents an early stage of impaired cognition in individuals who maintain the ability to independently perform most activities of daily living (Chun et al., 2021; Pinto et al., 2019). MCI may develop into dementia over time, but many MCI patients do not experience further cognitive declines and some revert to normal cognition (Ganguli et al., 2011).

Evidence is mounting that marriage in later life is associated with a reduced risk of dementia (Sachdev et al., 2013; Sommerlad et al., 2018) and MCI (Håkansson et al., 2009; Liu et al., 2019). Understanding the link between marital status and later-life dementia and MCI is highly relevant in light of population aging and the substantial changes in partnerships and living arrangements that have occurred over recent decades (Pesando et al., 2018), including increases in cohabitation and divorce and an increase in the number of people living alone (Smock & Schwartz, 2020). So far, much of the evidence linking marital status to dementia and MCI is based on cross-sectional data and relatively small samples, population-level studies remain relatively rare, and partnership status tends to refer to the participants’ current (and not earlier) situation (Sommerlad et al., 2018). Furthermore, many studies do not investigate both dementia and MCI. High quality nationwide longitudinal registry data, combined with careful clinical diagnosis of dementia and MCI, can give important insights in the relationship between marital histories and dementia risks and reduce selection problems (Waite & Gallagher, 2002). Our study contributes importantly to the literature by examining the association between six types of marital histories (unmarried, continuously divorced, intermittently divorced, widowed, continuously married, intermittently married) and later-life dementia and MCI using linked survey, clinical, and register data from Norway (*N* = 8706). We also examine whether number of children, health status, and social risk factors for dementia account for the association between marital histories and later-life dementia and MCI. We further observe marital status and health from earlier in life.

### Previous Research on Marital Status and Dementia and MCI

Recently, focus on how social relationships are related to later-life cognition has increased. It is estimated that approximately 3.5% of dementia cases are attributable to social isolation, which exceeds the proportion of cases attributable to physical inactivity and diabetes (Livingston et al., 2020). Marriage provides one of the most important sources of social contact and support in adulthood, and several studies have investigated the association between later-life marital status and later-life cognition. Findings from several countries report that being married and/or living with a partner appears to protect against dementia and MCI (Bickel & Cooper, 1994; Fratiglioni et al., 2000; Håkansson et al., 2009; Helmer et al., 1999; Liu et al., 2019) consistent with a broader literature documenting positive effects of marriage on health in general (Koball et al., 2010). A meta-analysis of 15 studies comprising *N* = 812,047 individuals (including one Swedish study of *N* = 750,129) found that people who were single their entire life or widowed had a higher risk of dementia than those who were married (Sommerlad et al., 2018). However, a challenge with many earlier cross sectional studies is that they rely on survey-based and self-reported assessments of marital status at a single time point and that the dementia/MCI diagnoses are registry-based. Many dementia cases go unreported in health registers (Gjøra et al., 2021), while surveys can involve selection at baseline and attrition affecting representativity (Anstey et al., 2010; Ruano et al., 2019). Some studies indicate that the protective effects of marriage against dementia risk are greater for men than for women (Najar et al., 2021), consistent with results showing that marriage benefits men more than women also for other dimensions of health (August & Sorkin, 2010; Kiecolt-Glaser & Newton, 2001).

### Why Does Marriage Matter: Potential Mediators

The relationship between marital histories and later-life health, including cognition, likely reflects both selection and causal effects (Mastekaasa, 1992; Murray, 2000). Marriage might affect social interaction, which in turn might build cognitive reserve, which reduces dementia risk (Guner et al., 2018a; Stern, 2012; Tosi & van den Broek, 2020; Tumin & Zheng, 2018). Divorce may cause social and financial stress, and involve conflict and health risk behaviors (Kiecolt-Glaser, 2018; Mata et al., 2018). Selection effects pertain to situations whereby people who marry and stay married may have characteristics that also protect them from later-life dementia and MCI. Research has found that the tendencies to marry and to divorce are influenced by heritability (Jerskey et al., 2010). Moreover, healthier persons are more likely to marry, while less healthy individuals are more likely to either remain single or become separated, divorced, or widowed (Martikainen et al., 2005). Men with low income and low education are less likely to marry than their peers with higher levels of income and education; in turn, low income and low educational attainment are also related to an increased risk of later-life dementia and MCI (Livingston et al., 2020; Nguyen et al., 2016). A Swedish study of individuals aged 77+ years from 1992 to 2014 suggests that low educational level and low cognitive function are both strongly associated with not being married and living alone over time (Fors et al., 2009).

Being married can have a causal, protective effect on later-life dementia and MCI (Jace & Makridis, 2021). For example, being married can raise often the amount of social interaction (Luo et al., 2022; Wadsworth, 2016), and having a spouse can also influence a number of health behaviors relevant for later-life cognition including alcohol consumption, smoking, physical inactivity, and diet (Liu & Waite, 2014). The positive effect of marriage on the risk of dementia may also be driven by better cardiovascular health among those who are married relative to those who are not married, also when economic factors and pre-marital health have been accounted for (Sommerlad et al., 2018; Umberson & Thomeer, 2020). Furthermore, marriage may affect mental health; currently married adults are less distressed than unmarried adults (DeMaris, 2018). Marriage also reduces depressive symptoms for both men and women, whereas being divorced has the opposite effect (Wilson & Oswald, 2005).

Fertility is another potential mediating factor that has not received much attention in relation to cognitive decline and marital status. Marital status is highly related to fertility; people who never marry are far less likely to have children (Holland, 2017; Lillard & Waite, 1993). In turn, having children may affect later-life cognition, albeit the effect could be in either direction (Sundström et al., 2016). Children may provide a sense of meaning, purpose in life, and encourage protective health behaviors (e.g., quitting smoking, physical exercise, regular check-ups) that, in turn, reduce the risk for dementia (Umberson & Thomeer, 2020). Children can be important sources of practical and emotional support, but may also be sources of mental distress. Selection may also play an integral role, as those who have children (at least among the male population in Norway) are positively selected in terms of early adulthood cognition (Bratsberg & Rogeberg, 2018). Importantly, the relationship between number of children and dementia may not be linear. A recent study found that having three or more children versus having two has a negative effect on late-life cognition (Bonsang & Skirbekk, 2022). A United States based study found no significant effect of number of children on the risk of dementia in men or women after correcting for sociodemographic and health characteristics (Gemmill & Weiss, 2020).

### Current Study

In the current study, we use a unique mix of Norwegian survey, clinical and register data to construct measures of marital history, and analyze how they differentially relate to a clinical diagnosis of dementia or MCI in later-life. Our population-level and longitudinal data are characterized by high response rates and many cases with clinically diagnosed dementia and MCI allow us to investigate this relationship in greater detail and with more accuracy than in many previous studies. Noting that the risk of MCI appears to be even higher among people who were unmarried in both mid- and later-life (Håkansson et al., 2009), we focus on marital histories in the 4th to the 6th decades of life as opposed to a one-time “snapshot” of marital status.

The longitudinal data set with a long time-period between exposure and outcome allows us to investigate the impact of midlife marriage and health status on old age dementia/MCI, which is highly relevant since pathology related to dementia starts decades before symptom onset and diagnosis.

Focusing on midlife marital histories is also in line with evidence that the beneficial effect of marriage on health is not evident before age 40 (Guner et al., 2018a). Consistent with earlier research, we hypothesize that the risk for dementia will be lower among those who are continuously married than among those who are divorced, widowed, or unmarried. Since MCI does not necessarily progress to dementia, we predict similar, but generally weaker associations between marital histories and MCI relative to dementia. Consistent with the broader literature on the effect of marriage on health, we further hypothesize that men benefit more than women from marriage in terms of reducing their risk of dementia and MCI. Finally, we hypothesize that the number of children and the health and social risk factors such as smoking, obesity, diabetes, hypertension, psychological stress, and having no close friends, may partially explain the relationship between marital histories and the cognitive outcomes considered.

## Methods

### Sample

The health data in the current study stem from the HUNT Study, a large, ongoing population-based health survey from the former Nord-Trøndelag County in Norway (Åsvold et al., 2022). Nord-Trøndelag is fairly representative of Norway (e.g., GDP and education levels are mid-range compared to the other Norwegian counties; Statistics Norway, 2021). All county inhabitants were invited to participate in four consecutive study waves: HUNT1, 1984–1986; HUNT2, 1995–1997; HUNT3, 2006–2008; and HUNT4, 2017–2019 (Åsvold et al., 2022; Gjøra et al., 2021).

The total sample comprised *N* = 8706 individuals born in the period 1931–1949 and aged 70+ who participated in clinical assessments of cognitive functioning and dementia (Gjøra et al., 2021). In this HUNT4 70+ study population, 90.3% participated in at least two earlier waves, and 87.3% had participated in all earlier waves (HUNT1–3). Just over half (53.2%) were women. The mean age of the sample was 76.6 years in 2018 (SD = 4.6, range = 70–88 years).

### Data Linkage

Every resident in Norway is assigned a unique national personal identification number, making it possible to seamlessly link register and survey records from the same person. In the current study, we linked sociodemographic information on age, sex, education, marital status, and number of children from the Norwegian national population registry with the health survey and clinical diagnostic data from the HUNT studies.

### Dementia and MCI Diagnoses

Clinical dementia and MCI diagnoses were obtained from HUNT4 70+ (2017–2019). All participants in HUNT4 70+ underwent a comprehensive clinical evaluation conducted by trained personnel at a field station, the participant’s home, or a long-term care facility. An interview with the next of kin was conducted for all long-term care residents and whenever dementia and/or MCI was suspected. A range of instruments was used, covering cognition, daily functioning, neuropsychiatric symptoms, subjective cognitive decline, symptom debut, and course of the condition. The cognitive assessment protocol comprised the Montreal Cognitive Assessment scale (MoCA) (Julayanont et al., 2015; Nasreddine et al., 2005) and the Word List Memory Task from the Consortium to Establish a Registry of Alzheimer’s Disease (Moms et al., 1989) as well as a trail making test. For participants with severe dementia, the Severe Impairment Battery-8 (Schmitt et al., 2013) was used instead of MoCA. Two independent clinicians used the obtained information to diagnose each participant according to the DSM-5 criteria. Diagnosis was either *no cognitive impairment*, *mild neurocognitive disorder* (corresponding to MCI), or *dementia* (Gjøra et al., 2021).

### Main Exposure Variable: Marital History

We obtained data on marital status from the population registries for each year ranging from 1975–2017. Marital status was defined as unmarried (including both single and cohabitating), divorced/separated, married, or widowed. We used the yearly data to define participants’ marital histories between age 44 and 68 years, that is, a 24-year age span (see the Lexis diagram in Figure S1). Individuals who consistently maintained the same marital status for the whole 24-year period were defined as either never married, continuously divorced/separated (denoted divorced), continuously widowed, or continuously married. Individuals who changed marital status were grouped according to their predominant status and labeled “intermittent.” For example, a person who was married between ages 44 and 50 and divorced thereafter was defined as “intermittently divorced.” Few individuals were defined as intermittently unmarried or intermittently widowed. We therefore merged the intermittently and continuously unmarried and widowed groups, respectively. The final marital histories variable consisted of six groups: unmarried, continuously divorced, intermittently divorced, widowed, continuously married, intermittently married. Notably, ≤1.0% (depending on the specific HUNT wave) of those defined as unmarried were cohabitating with a partner according to a self-report. In a sensitivity analysis, we restricted the participants' marital histories between age 44 and 60 years, that is, a 17-year age span to understand the extent to which the timing of marriage mattered.

### Confounding Variables: Age, Sex, and Education

To account for confounding, we statistically controlled for the registry-based variables age, sex, and education. To simplify the analyses, we defined three age groups (69–74, 75–79, and 80+ years) and three educational levels (primary, secondary, tertiary). We also investigated whether the results were moderated by sex, through using sex as an interaction term in the analyses.

### Potential Mediators

Number of children was available from the Norwegian population registry and was categorized as 0, 1, 2, 3, or 4+ children.

#### Health and Social Risk Factors

Data on seven risk factors for dementia and MCI were available in HUNT: smoking, hypertension, obesity, physical inactivity, diabetes, mental distress, and no close friends. We defined a series of dummy variables having (no/yes). *Smoking* was defined as being a self-reported current or former smoker at least once in HUNT1–3*. Hypertension* was defined as having systolic blood pressure ≥ 140 mmHg and/or diastolic blood pressure ≥ 90 mmHg (based on the mean of two measurements in HUNT1, and three measurements in HUNT2 and 3) at least once in HUNT1–3. *Obesity* was defined as having a body mass index (kg/m^2^) of ≥ 30 at least once in HUNT1–3, based on measured height and weight. *Physical inactivity* was defined as not performing the minimum recommended physical activity of at least 30 min per day, according to the classification on the physical activity questionnaire in HUNT (Kurtze et al., 2008) at least once in HUNT1–3. *Diabetes* was defined as having an elevated blood glucose level (≥11.1 mmol/L) and, or reporting having diabetes at least once in HUNT1-3. *Mental distress* was defined as scoring at least 2.15 on the CONOR-Mental health index (Næss et al., 2008), or reported seeking help for mental health problems or ever having mental health problems in HUNT2 or 3 (not included in HUNT1). *No close friends* was defined as reporting having no close friends in either HUNT2 or 3 (not included in HUNT1).

### Statistical Analysis

We used STATA (SE version 17) for all analyses (Statacorp, 2022). We used inverse probability weights (IPW) to account for non-response in HUNT4 70+ and to correct for bias due to skewed participation by age, sex, and educational level. We had access to a registry-based dataset with the variables age, sex, education, and participation for all those invited to participate in the HUNT4 70+ study (*N* = 19,463). Using this dataset, we predicted the probability of participation using a logistic regression model with a dichotomous outcome variable (participated: yes/no) and sex, age, and education (primary, secondary, tertiary) as predictors. Just over half (*N* =9930, 51%) of the 19,463 persons invited participated in HUNT4 70+survey. Participation was higher among women, in the youngest age group, and in the higher educational groups. The inverse of the probability of participation was used as IPW, providing 36 distinct weights (Table S1), which were merged to the participants. As our analysis was restricted to those younger than age 45 in 1975, the initial year of marital status information in the registry, our study population was limited to those born during 1931–1949 (17,009 invited, 8706 participated).

Data from the registers (age, sex, education, marital status, number of children) was available for all participants. Missing values for the health and social risk factors from HUNT were imputed using multiple imputation. Year of birth, sex, number of children, and education were used as non-missing variables in the prediction. Logistic regression was used to impute 30 datasets. The number and proportion of imputed data were: smoking (n = 416, 5.2%), obesity (n = 351, 4.0%), hypertension (n = 347, 4.0%), physical inactivity (n = 590, 6.8%), diabetes (n = 341, 3.9%), mental distress (n = 622, 7.1%), no close friends (n = 1055, 12.1%).

To assess the association between marital history and dementia/MCI, we used multinomial logistic regression to estimate relative-risk ratios (RRR) using continuously married (the most common trajectory, see below) as the reference. No cognitive impairment served as the base outcome and dementia and MCI served as the two other possible outcome categories. Although MCI is considered an intermediate stage between normal cognition and dementia, according to a meta-analysis most people with MCI will not progress to dementia even after 10 years of follow-up (Shiri-Feshki, 2009). We therefore used multinomial as opposed to ordinal logistic regression.

To investigate whether the association between marital status and cognitive outcomes were mediated by the number of children, physical health risk factors, or mental health, we applied the classical difference method by fitting regression models with and without the mediators. If the effect of marital status trajectories were reduced after adjustment, this would indicate mediation. In addition, guided by the main results from this basic approach, and to allow for nonlinear relationships, we performed a causal mediation analysis with the Stata module PARAMED using binary exposures (continuously married versus unmarried, continuously married versus continuously divorced) and binary mediators one-by-one (Emsley & Liu, 2013). The size of the mediation was estimated using nie/(nie+nde), where nie is the log of the odds ratio for the indirect effect, and nde is the log of the odds ratio for the direct effect. There were no significant interactions between the main exposure (marital history) and the mediators, so the model did not have to take this into account. IPW and multiple imputation were combined to give a doubly robust estimator (Seaman et al., 2012).

To examine whether the association between marital history and dementia/MCI differed according to sex, we (a) included marital history by sex interaction terms, and (b) stratified the analyses according to sex. Sensitivity analyses were performed without IPW and without imputation, through running the analyses on the complete case data set without any missing values (n = 7451/8706, 86%).

In an additional set of analyses, we dichotomized the outcome variable into dementia versus MCI or no impairment, and we used logistic regression with IPW to calculate odds ratios. Using these odds ratios combined with prevalence estimates of marital history groups, we calculated the *population attributable fraction* (PAF), that is, the reduction in dementia cases that would have potentially occurred in a counterfactual scenario where all participants were continuously married.

## Results

A total of 11.6% participants were diagnosed with dementia and 35.3% were diagnosed with MCI. The most prevalent dementia subtype was Alzheimer’s disease (57%), followed by vascular dementia (10%) (Gjøra et al., 2021), which is close to the distribution of dementia reported in other similarly-aged populations (World Health Organization, 2021). Most participants were continuously married between ages 44 and 68 (70.5%); the rest were intermittently married (11.3%), unmarried (4.6%), continuously divorced (3.9%), intermittently divorced (5.3%), or widowed (4.3%).

Table 1 displays the characteristics of the sample by diagnosis. The associations between age, sex, education, number of children, the health risk factors, mental distress, and having no close friends on the one hand and dementia and MCI on the other hand were consistent with previous research (see Table 1): Namely, older age, female sex, lower education, smoking, hypertension, obesity, diabetes, physical inactivity, mental distress, and no close friends were each associated with a higher risk for dementia and MCI.

**Table 1.** Sample Characteristics.

	Cognitive diagnosis					
	n	No impairment (%)	n	MCI %	n	Dementia %
Total	4621	(53.1)	3071	(35.3)	1014	(11.6)
Registry-based variables						
Sex						
Women	2543	(54.9)	1533	(33.1)	554	(12.0)
Men	2078	(51.0)	1538	(37.7)	460	(11.3)
Age 2018, years						
69–74	2080	(58.1)	1295	(36.2)	207	(5.8)
75–79	1508	(53.9)	1011	(36.1)	278	(9.9)
80+	1033	(44.4)	765	(32.9)	529	(22.7)
Marital history 44–68 years						
Unmarried	179	(44.3)	168	(41.6)	57	(14.1)
Always divorced	174	(51.6)	122	(36.2)	41	(12.2)
Predominantly divorced	253	(54.9)	150	(32.5)	58	(12.6)
Widow	193	(51.1)	136	(36.0)	49	(13.0)
Always married	3319	(54.0)	2134	(34.8)	688	(11.2)
Predominantly married	503	(51.1)	361	(36.6)	121	(12.3)
Number of children						
0	249	(45.5)	211	(38.6)	87	(15.9)
1	283	(45.8)	239	(38.7)	96	(15.5)
2	1655	(55.5)	1051	(35.3)	275	(9.2)
3	1592	(55.5)	981	(34.2)	294	(10.3)
4+	842	(49.7)	589	(34.8)	262	(15.5)
Education						
Compulsory	817	(40.0)	827	(40.5)	399	(19.5)
Secondary	2561	(53.7)	1713	(35.9)	491	(10.3)
Tertiary	1243	(65.5)	531	(28.0)	124	(6.5)
Health and social risk factors HUNT1–3						
Ever smoker						
No	1909	(56.4)	1109	(32.8)	364	(10.8)
Yes	2518	(51.3)	1814	(37.0)	576	(11.7)
Ever hypertension						
No	1566	(57.1)	943	(34.4)	235	(8.6)
Yes	2882	(51.3)	2007	(35.7)	726	(12.9)
Ever obese						
No	3356	(54.0)	2176	(35.0)	679	(10.9)
Yes	1093	(51.0)	772	(36.0)	279	(13.0)
Ever physically inactive						
No	1708	(57.4)	978	(32.9)	288	(9.7)
Yes	2636	(51.3)	1875	(36.5)	631	(12.3)
Ever diabetes						
No	4217	(53.9)	2729	(34.9)	871	(11.1)
Yes	234	(42.7)	224	(40.9)	90	(16.4)
Ever mental problems						
No	3427	(54.5)	2218	(35.3)	646	(10.3)
Yes	913	(50.9)	640	(35.7)	240	(13.4)
No close friends						
No	3718	(54.3)	2408	(35.2)	722	(10.5)
Yes	415	(51.7)	294	(36.6)	94	(11.7)

*Note*: Smoking, hypertension, obesity, and diabetes are based on HUNT1–3. Mental distress and no close friends are based on HUNT2–3. Number of participants (%) by diagnosis, HUNT4 70+ cohort born 1931–49. *N* = 8706.

Table 2 displays the characteristics of the sample by marital history. Notably, the prevalence of dementia was higher among the unmarried (14.1%) than the continuously married group (11.2%). The health and social risk factors also differed across the marital trajectory groups. For instance, 76.1% were defined as smoking in the continuously divorced group, compared with 57.8% in the continuously married group. The majority (77.0%) of the unmarried group were childless, compared with just 1.9% of the continuously married and 2.7% of the continuously divorced.

**Table 2.** Characteristics by Marital History in the HUNT4 70+ Cohort Born 1931–49 (*N* = 8706).

	Unmarried		Continuously divorced		Intermittently divorced		Widowed		Continuously married		Intermittently married	
Total	404	100.0 (%)	337	100.0 (%)	461	100.0 (%)	378	100.0 (%)	6141	100.0 (%)	985	100.0 (%)
Registry-based variables												
Women Age (years)	142	35.1	215	63.8	230	49.9	323	85.4	3139	51.1	581	59.0
69–74	192	47.5	208	61.7	226	49.0	130	34.4	2408	39.2	418	42.4
75–79	109	27.0	90	26.7	170	36.9	120	31.7	2005	32.6	303	30.8
80+	103	25.5	39	11.6	65	14.1	128	33.9	1728	28.1	264	26.8
Education												
Primary	104	25.7	98	29.1	99	21.5	113	29.9	1385	22.6	244	24.8
Secondary	219	54.2	180	53.4	234	50.8	209	55.3	3408	55.5	515	52.3
Tertiary	81	20.0	59	17.5	128	27.8	56	14.8	1348	22.0	226	22.9
Number of children												
0	311	77.0	9	2.7	20	4.3	17	4.5	114	1.9	76	7.7
1	62	15.3	40	11.9	40	8.7	25	6.6	370	6.0	81	8.2
2	25	6.2	132	39.2	161	34.9	123	32.5	2221	36.2	319	32.4
3	5	1.2	103	30.6	162	35.1	125	33.1	2188	35.6	284	28.8
4+	1	0.2	53	15.7	78	16.9	88	23.3	1248	20.3	225	22.8
Variables from HUNT1-3												
Smoking	189	51.1	232	76.1	281	69.7	209	56.8	3436	57.8	561	62.3
Hypertension	275	72.8	177	56.7	236	57.7	247	66.6	4074	68.1	606	66.6
Obesity	99	26.3	75	24.0	98	24.0	105	28.2	1532	25.6	235	25.8
Physical inactivity	226	61.9	192	65.1	221	58.5	254	69.8	3695	63.2	554	63.8
Diabetes	30	7.9	13	4.2	23	5.6	24	6.5	406	6.8	52	5.7
Mental distress	81	23.0	105	36.1	149	38.5	90	25.6	1124	19.3	244	27.9
No close friends	51	15.6	30	11.1	33	9.2	21	6.3	597	10.8	71	8.5

*Note*: Smoking, hypertension, obesity, and diabetes are based on HUNT1–3. Mental distress and no close friends are based on HUNT2–3.

### Higher Risk of Dementia for the Unmarried and Continuously Divorced in MidLife

**Figure 1.**
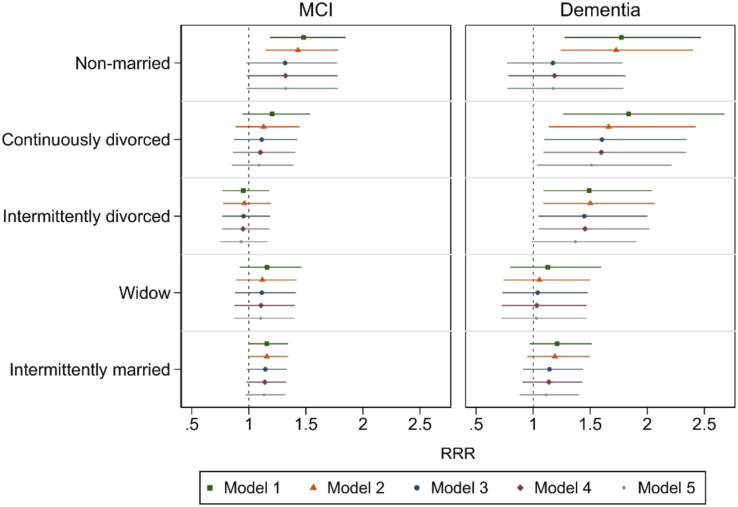
Relative risk ratios (RRRs) for MCI or dementia estimated in multinomial logistic regression. Main exposure is marital status trajectories during age 44-68 years, with continuously married as reference line at 1.0. Age range is 70-87 years at HUNT4 70+. Analyses are weighted for non-response (IPW) and missing values are imputed using multiple imputation (MI), chained logit (30 samples). N=8706 (see numbers in Table S2). Model 1 was adjusted for age and sex; Model 2 was additionally adjusted for education; Model 3 was additionally adjusted for number of children; Model 4 was additionally adjusted for smoking, hypertension, obesity, physical inactivity, and diabetes; Model 5 was additionally adjusted for mental distress and no close friends.

The full results of the multinomial logistic regression model are available in Table S2. Figure 1 displays the RRR of the marital history groups in each model using the continuously married as the reference group. After adjusting for age, sex, and education, the risk of dementia was higher for the unmarried (RRR = 1.73; 95% CI: 1.24, 2.40), continuously divorced (RRR = 1.66; 95% CI: 1.14, 2.43), and intermittently divorced (RRR = 1.50; 95% CI: 1.09, 2.06). The widowed and intermittently married groups had the same risk for dementia and MCI as the continuously married. In general, marital history was less associated with MCI than with dementia (Figure 1, Table S2). After adjusting for age, sex, and education, the risk of MCI was only higher among the unmarried (RRR = 1.43; 95% CI: 1.15, 1.78) relative to the continuously married. None of the other marital history groups differed in MCI risk compared to the continuously married group after controlling for age, sex, and education.

In the counterfactual scenario, where all participants had the same risk of receiving a dementia diagnosis as in the continuously married group, 6.0% of all dementia cases could be avoided (PAF = 6.0; 95% CI: 2.3–9.5, adjusted for age and sex) if everyone were always married.

### Number of Children, Health and Social Risk Factors Account for the Relationships between Marital History and Cognitive Impairment

The elevated risk of dementia for the unmarried group was no longer significant after adjusting for number of children (RRR = 1.17; 95% CI: .77–1.78). The elevated risk of dementia for the continuously divorced group was robust against adjustment for number of children, the physical health-related risk factors, and mental distress and no close friends. The elevated risk of dementia for the intermittently divorced group was robust to adjustment for number of children and the physical health risk factors, but did not statistically differ from the continuously married group (RRR = 1.37; 95% CI .98–1.90) when the model was adjusted for mental distress and no close friends. The unmarried group had a higher risk of MCI than the continuously married group, even after adjusting for age, sex, and education. Additionally, controlling for number of children rendered the difference between the unmarried and continuously married groups insignificant.

### No Evidence of Sex Interaction between Dementia/MCI Risks and Marital Histories

Overall, the general pattern of the relationships between marital histories and dementia/MCI risk was similar for men and women, and there was no evidence of an interaction between marital histories and sex for dementia (*p* = .76) or MCI (*p* = .26) (Figure S2).

### Why Do The Unmarried Have Increased Dementia Risk? A Causal Mediation Analysis

The regression analyses above suggested that the increased dementia risk among the unmarried was mediated by their number of children (Table S2, Model 3), while none of the other covariates (Model 4 + 5) appeared to affect the confounder adjusted RRRs in Model 2 for either dementia or MCI. To investigate why the unmarried had an elevated risk for dementia, we conducted a causal mediation analysis. Participants with MCI were removed from the analyses, and dementia (yes/no) was the outcome variable. Furthermore, marital history was restricted to unmarried (exposed) and continuously married (non-exposed). Number of children was coded (0/1+) and was a binary mediator. We also included diabetes (yes/no), hypertension (yes/no), ever smoker (yes/no), ever obese (yes/no), ever physical inactive (yes/no), ever no close friends (no/yes), and mental distress (yes/no). Except for the number of children, there was no evidence that any of these variables were mediators. The marginal total effect of being unmarried was OR = 1.74 (95% CI: 1.20, 2.34), of which 1.24 was a direct effect and 1.39 was an indirect effect. Thus, 60% of the total effect of being unmarried was indirectly associated with dementia via being childless, while 40% were due to other factors associated with being non-married, and none of these factors were related to ever diabetes, ever hypertension, ever smoker, ever obese, ever physically inactive, ever no close friends, or ever mental distress.

### Sensitivity Analysis

To assess if the results would differ if we changed the observational window, we shifted the participants’ marital histories from age 44–68 years to 44–60 years. Consequently, the percentage widowed declined from 4.3% to 2.9%, while the continuously married increased from 70.5% to 75.3%. The unmarried group was identical in the two settings. Regression results were robust and similar for the two age windows. More specifically, in age- and sex-adjusted results, continuously divorced had RRR 1.71 with the age-window 44–60 versus 1.84 previously using the age window 44–68 (Table S2), both being significant. For the unmarried, the RRR changed from 1.77 to 1.70; for widowed the RRR changed from 1.13 to 1.26 (both non-significant). Thus, the sensitivity check suggests that the age windows 44–60 and 44–68 capture the same underlying effects and the choice of age window does not affect our main findings or conclusions. Note that divorce is less likely to occur after age 68 years. Divorce rates in Norway were 1.4 per 1000 men in 2021 among those above age 70 years, compared to 15.4 among those in their 40s (the age group that has the highest divorce rate) (Statistics Norway, 2021).

## Discussion

Our findings add to the burgeoning evidence that marriage is associated with a lower risk for dementia and MCI in later life. The higher risk of dementia among the unmarried and divorced groups in our study corroborates evidence from other countries including Finland (Håkansson et al., 2009), France (Helmer et al., 1999), and the United States (Liu et al., 2019), and a recent meta-analysis (where widows, but not divorced, were at increased dementia risk) (Sommerlad et al., 2018). We estimate that, had all participants been continuously married (and shared the same underlying somatic and mental health plus social characteristics of those who marry), 6% of the dementia cases in our study would not have occurred. This is a considerable reduction and is equivalent to the proportion of dementia cases accounted for by smoking and obesity combined, as reported by the Lancet dementia commission in 2020 (Livingston et al., 2020). However, it is not clear to which extent or how marriage can be an effective policy lever to reduce MCI and/or dementia.

In the Lancet report, partnership was used as a proxy measure for social contact in older age and was estimated to account for 4% of dementia cases. Our slightly higher estimate could be due to the fact that our marital history variable was registry-based and covered a long time-span from adult age into older age, as opposed to only a snapshot in time which is usually used (Livingston et al., 2020; Sommerlad et al., 2018). We did not observe any significant interaction between marital history and sex, which may suggest that staying married throughout adulthood is as beneficial for women as it is for men. This could potentially be linked to Norway being a highly gender-equal society (Kitterød & Nadim, 2020; Vikat & Jones, 2014).

Our results showed that the unmarried had a 43% higher risk of MCI than the continuously married after adjusting for age, sex, and education. We further found that participants who were continuously divorced had an 66% elevated risk of being diagnosed with dementia in later-life compared to participants who were continuously married, after adjusting for age, sex, and education, contrasting with the findings from (Sommerlad et al., 2018) who found no significant relationship between dementia and divorce. Participants in the intermittently divorced and unmarried groups also had elevated risks of dementia compared to continuously married participants. The elevated dementia risk for those with a history of divorce was only weakly mediated by the health and social risk factors. However, particularly noteworthy is that number of children accounted for 60% of the relationship between being unmarried and dementia risk; yet, given the strong correlation between having children and marriage in the studied cohort, our analyses cannot identify whether having (no) children or not being married is the primary mechanism through which the unmarried in our study are most at risk of dementia.

### Implications

Our study draws further attention to marital histories as a predictor of later-life cognitive impairment, and the potential mediating roles of having children, health, and social risk factors. Information on the link between marital status and later-life cognition could be useful for individuals as they consider the benefits and costs of different family forms, although we highlight that our results do not allow us to identify causal effects. It is also uncertain how ongoing changes in the prevalence of particular family constellations (e.g., increases in divorce and cohabitation along cohort lines) may change the relationship between marital status trajectories and later-life cognitive impairment. Norwegian data have suggested that the selection of people—particularly men—into marriage based on grip strength has increased for younger cohorts (Skirbekk et al., 2018), suggesting that the relationship between being unmarried and health may be even stronger in younger cohorts.

Our finding that 6.0% of all dementia cases are related to being unmarried in midlife suggests that marital choices can be of importance for overall dementia prevalence. In light of ongoing societal changes in partnering and marriage dynamics, more attention should be given to family constellations over the life course as these correlate with dementia.

The results of our study have implications for healthcare service planning. The decrease in marriage and increase in divorce (Cherlin, 2010; SSB, 2021; Zahl-Olsen et al., 2019) could have implications for future dementia (and MCI) incidence and hence future health care provision. Divorce is still very rare in older adults (65+) in Norway, but it is rising. Divorce rates among adults 70 + are less than a tenth of those observed among adults in their 40s (Statistics Norway, 2022).

One could seek to identify preventive measures to attenuate the risk of dementia among people who are not continuously married. Governments and social planners need to be aware of the increased risk among those who are single and may consider offering alternative services and social activity initiatives to increase the level of social contact. A greater prevalence of dementia among those who are single suggests that many will need healthcare service earlier. In spite of the fact that poor cognition predicts a lower capacity to live independently (Tangen et al., 2020), an increasing number of individuals with cognitive impairment do so (Portacolone et al., 2019). The current healthcare model increasingly emphasizes home-based care (Næss et al., 2008).

### Strengths, Limitations, and Suggestions for Future Research

The strengths of our study include the large cohort of older adults drawn from the general population of Nord-Trøndelag, longitudinal and multiple measurements of health and social risk factors for dementia, the rich registry-based data and the thorough clinical assessments and diagnoses of dementia and MCI. We also use sample weighting to account for non-participation. We had extensive demographic, socioeconomic, health, and social information from the earlier HUNT surveys (preceding the HUNT4 data by up to 35 years) and information from population registers (preceding the dementia and MCI diagnoses by up to 44 years). This renders our data unique compared to other available data with clinically-diagnosed dementia cases. The longitudinal data set-up and the long time-period between exposure and outcome using high quality Norwegian population-level data make this study unique. The fact that we can follow health and social trajectories allows us to investigate a period of life where midlife status is important and not usually investigated.

Our analysis does not allow us to draw conclusions about the causal effects of marital histories on dementia or MCI. We were also unable to include information on genetic factors. Also, the number of individuals who were unmarried, divorced, or widowed was relatively low. The unmarried group also included a small proportion of people who were cohabitating with a partner (in our sample ≤1%), which may have slightly “diluted” its effect. Finally, we identified marital status from ages 44–68, and observed dementia and MCI status at age 70+. In some participants, marital status was thus measured only two years prior to the diagnosis (i.e., those who were aged 70 at the time of the diagnosis), while marital status was measured up to 20 years prior to diagnosis in other participants (i.e., those who were aged 88 at the time of diagnosis). This means that the exposure-duration of being divorced, widowed, married, or unmarried was longer for some people than for others. However, results from the sensitivity analysis in which we restricted marital histories to ages 44–60 did not differ significantly from the findings from the main analysis.

More research is needed to understand the mechanisms linking marital histories and cognitive impairment. In particular, we found that the continuously divorced had an increased risk for dementia even after adjusting for number of children and all of the considered health and social risk factors. Other factors appear to mediate the relationship between being continuously divorced and cognitive impairment than the factors investigated in the current study. Given ongoing changes in family formation patterns and living constellations, future research should also investigate cohort differences in the relationship between marital status/history and later-life dementia and MCI. More research should also be carried out to investigate how changes in fertility (childbearing) affect cognition. For instance, there have been changes in the timing of childbearing (parents are older when having children) and in terms of parity progression (fewer parents have a high number of children), as well as rising shares of people foregoing childbearing altogether. How such changes are related to cognitive outcomes will be addressed in future work.

## Supplemental Material

Supplemental Material - Marital Histories and Associations With Later-Life Dementia and Mild Cognitive Impairment Risk in the HUNT4 70+ Study in Norway Supplemental Material for Marital Histories and Associations With Later-Life Dementia and Mild Cognitive Impairment Risk in the HUNT4 70+ Study in Norway by Vegard Skirbekk, Asta Håberg, Ekaterina Zotcheva, Bo Lars Engdahl, Steinar Krokstad, Hans-Peter Kohler, Astanand Jugessur, PhD, Bernt Bratsberg, Jordan Weiss, PhD, Catherine Bowen^,^ Sarah Tom, PhD, Jennifer Harris, PhD, Yaakov Stern, PhD, Geir Selbæk, and Heine Strand, PhD in Journal of Aging and Health

## ORCID iDs

Vegard Skirbekk https://orcid.org/0000-0002-1647-3246

Asta Håberg https://orcid.org/0000-0002-9007-1202

Catherine E. Bowen https://orcid.org/0000-0003-3887-2691

Geir Selbæk https://orcid.org/0000-0001-6511-8219

Hans-Peter Kohler https://orcid.org/0000-0003-0639-4349

Jennifer R. Harris https://orcid.org/0000-0003-3249-8918

Steinar Krokstad https://orcid.org/0000-0002-2932-6675

Sarah E. Tom https://orcid.org/0000-0003-0094-5138

Yaakov Stern https://orcid.org/0000-0001-7542-3241

Bjørn Heine Strand https://orcid.org/0000-0003-4385-8886

## References

[bibr1-08982643221131926] Anstey K. J. Burns R. A. Birrell C. L. Steel D. Kiely K. M. Luszcz M. A. (2010). Estimates of probable dementia prevalence from population-based surveys compared with dementia prevalence estimates based on meta-analyses. BMC Neurology, 10(1), 1–12. 10.1186/1471-2377-10-62 20646331 PMC2912843

[bibr2-08982643221131926] Åsvold B. O. Langhammer A. Rehn T. A. Kjelvik G. Grøntvedt T. V. Sørgjerd E. P. Fenstad J. S. Heggland J. Holmen O. Stuifbergen M. C. Brumpton B. M. Skjellegrind H. K. Thingstad P. Sund E. R. Selbæk G. Mork P. J. Rangul V. Hveem K. Vikjord S. A. A. , …, & Krokstad S. (2022). Cohort profile update: The HUNT study, Norway. International Journal of Epidemiology, 1–12. 10.1093/ije/dyac095 35578897 PMC9908054

[bibr3-08982643221131926] August K. J. Sorkin D. H. (2010). Marital status and gender differences in managing a chronic illness: The function of health-related social control. Social Science & Medicine, 71(10), 1831–1838. 10.1016/j.socscimed.2010.08.022 20889249 PMC2967659

[bibr4-08982643221131926] Bickel H. Cooper B. (1994). Incidence and relative risk of dementia in an urban elderly population: Findings of a prospective field study. Psychological Medicine, 24(1), 179–192. 10.1017/s0033291700026945 8208883

[bibr115-08982643221131926] Bonsang, E., & Vegard S . (2002). “Does Childbearing Affect Cognitive Health in Later Life? Evidence From an Instrumental Variable Approach.” Demography 59(3) 975–994. 10.1215/00703370-9930490 PMC10539463 35471229

[bibr5-08982643221131926] Bratsberg B. Rogeberg O. (2018). Flynn effect and its reversal are both environmentally caused. Proceedings of the National Academy of Sciences, 115(26), 6674–6678. 10.1073/pnas.1718793115 PMC6042097 29891660

[bibr6-08982643221131926] Cherlin A. J. (2010). Demographic trends in the United States: A review of research in the 2000s. Journal of Marriage and Family, 72(3), 403–419. 10.1111/j.1741-3737.2010.00710.x 22399825 PMC3293163

[bibr7-08982643221131926] Chun C. T. Seward K. Patterson A. Melton A. MacDonald-Wicks L. (2021). Evaluation of available cognitive tools used to measure mild cognitive decline: A scoping review. Nutrients, 13(11), 3974. 10.3390/nu13113974 34836228 PMC8623828

[bibr8-08982643221131926] DeMaris A. (2018). Marriage advantage in subjective well-being: Causal effect or unmeasured heterogeneity? Marriage & Family Review, 54(4), 335–350. 10.1080/01494929.2017.1359812 32382201 PMC7205226

[bibr9-08982643221131926] Emsley R. Liu H. (2013). Paramed: Stata module to perform causal mediation analysis using parametric regression models.

[bibr10-08982643221131926] Fors S. Lennartsson C. Lundberg O. (2009). Childhood living conditions, socioeconomic position in adulthood, and cognition in later life: Exploring the associations. Journals of Gerontology Series B: Psychological Sciences and Social Sciences, 64(6), 750–757. 10.1093/geronb/gbp029 19420323

[bibr11-08982643221131926] Fratiglioni L. Wang H.-X. Ericsson K. Maytan M. Winblad B. (2000). Influence of social network on occurrence of dementia: A community-based longitudinal study. Lancet, 355(9212), 1315–1319. 10.1016/s0140-6736(00)02113-9 10776744

[bibr12-08982643221131926] Ganguli M. Snitz B. E. Saxton J. A. Chang C.-C. H. Lee C.-W. Vander Bilt J. Hughes T. F. Loewenstein D. A. Unverzagt F. W. Petersen R. C. (2011). Outcomes of mild cognitive impairment by definition: A population study. Archives of Neurology, 68(6), 761–767. 10.1001/archneurol.2011.101 21670400 PMC3135309

[bibr13-08982643221131926] Gates N. J. Vernooij R. W. Di Nisio M. Karim S. March E. Martinez G. Rutjes A. W. (2019). Computerised cognitive training for preventing dementia in people with mild cognitive impairment. Cochrane Database of Systematic Reviews, 3(3), CD012279. 10.1002/14651858.cd012279.pub2 30864747 PMC6415132

[bibr14-08982643221131926] Gemmill A. Weiss J. (2020). The relationship between fertility history and incident dementia in the US Health and Retirement Study. The Journals of Gerontology: Series B, 77(6), 1118–1131. 10.1093/geronb/gbab183 10.1093/geronb/gbab183 34614155

[bibr15-08982643221131926] Gjøra L. Heine Strand B. Bergh S. Borza T. Brækhus A. Engedal K. Johannessen A. Kvello-Alme M. Krokstad S. Matthews F. E. Myrstad C. Skjellegrind H. Thingstad P. Aakhus E. Aam S. Selbæk G. Livingston G. (2021). Current and future prevalence estimates of mild cognitive impairment, dementia, and its subtypes in a population-based sample of people 70 years and older in Norway: The HUNT Study. Journal of Alzheimer's Disease (Preprint), 79(3), 1–14. 10.3233/jad-201275 PMC7990439 33427745

[bibr16-08982643221131926] Guner N. Kulikova Y. Llull J. (2018a). Marriage and health: Selection, protection, and assortative mating. European Economic Review, 104, 138–166. 10.1016/j.euroecorev.2018.02.005 33132405 PMC7597938

[bibr18-08982643221131926] Håkansson K. Rovio S. Helkala E.-L. Vilska A.-R. Winblad B. Soininen H. Nissinen A. Mohammed A. H. Kivipelto M. (2009). Association between mid-life marital status and cognitive function in later life: Population based cohort study. Bmj, 339, b2462. 10.1136/bmj.b2462 19574312 PMC2714683

[bibr19-08982643221131926] Helmer C. Damon D. Letenneur L. Fabrigoule C. Barberger-Gateau P. Lafont S. Fuhrer R. Antonucci T. Commenges D. Dartigues J. F. Orgogozo J. (1999). Marital status and risk of Alzheimer’s disease: A French population-based cohort study. Neurology, 53(9), 1953–1953. 10.1212/wnl.53.9.1953 10599764

[bibr20-08982643221131926] Holland J. A. (2017). The timing of marriage vis-à-vis coresidence and childbearing in Europe and the United States. Demographic Research, 36(1), 609–626. 10.4054/demres.2017.36.20

[bibr21-08982643221131926] Jace C. E. Makridis C. A. (2021). Does marriage protect mental health? Evidence from the COVID-19 pandemic. Social Science Quarterly, 102(6), 2499–2515. 10.1111/ssqu.13063 PMC8662208 34908604

[bibr22-08982643221131926] Jerskey B. A. Panizzon M. S. Jacobson K. C. Neale M. C. Grant M. D. Schultz M. Eisen S. A. Tsuang M. T. Lyons M. J. (2010). Marriage and divorce: A genetic perspective. Personality and Individual Differences, 49(5), 473–478. 10.1016/j.paid.2010.05.007 20729979 PMC2923822

[bibr23-08982643221131926] Julayanont P. Tangwongchai S. Hemrungrojn S. Tunvirachaisakul C. Phanthumchinda K. Hongsawat J. Suwichanarakul P. Thanasirorat S. Nasreddine Z. S. (2015). The montreal cognitive assessment—Basic: A screening tool for mild cognitive impairment in illiterate and low-educated elderly adults. Journal of the American Geriatrics Society, 63(12), 2550–2554. 10.1111/jgs.13820 26648041

[bibr24-08982643221131926] Kiecolt-Glaser J. K. (2018). Marriage, divorce, and the immune system. American Psychologist, 73(9), 1098–1108. 10.1037/amp0000388 30525786 PMC6293993

[bibr25-08982643221131926] Kiecolt-Glaser J. K. Newton T. L. (2001). Marriage and health: His and hers. Psychological Bulletin, 127(4), 472–503. 10.1037/0033-2909.127.4.472 11439708

[bibr26-08982643221131926] Kitterød R. H. Nadim M. (2020). Embracing gender equality. Demographic Research, 42(14), 411–440. 10.4054/demres.2020.42.14

[bibr27-08982643221131926] Koball H. L. Moiduddin E. Henderson J. Goesling B. Besculides M. (2010). What do we know about the link between marriage and health? Sage Publications Sage CA.

[bibr28-08982643221131926] Kurtze N. Rangul V. Hustvedt B.-E. Flanders W. D. (2008). Reliability and validity of self-reported physical activity in the Nord-Trøndelag Health Study—HUNT 1. Scandinavian Journal of Public Health, 36(1), 52–61. 10.1177/1403494807085373 18426785

[bibr29-08982643221131926] Lillard L. A. Waite L. J. (1993). A joint model of marital childbearing and marital disruption. Demography, 30(4), 653–681. 10.2307/2061812 8262286

[bibr30-08982643221131926] Lissek V. Suchan B. (2021). Preventing dementia? Interventional approaches in mild cognitive impairment. Neuroscience & Biobehavioral Reviews, 122, 143–164. 10.1016/j.neubiorev.2020.12.022 33440197

[bibr31-08982643221131926] Liu H. Waite L. (2014). Bad marriage, broken heart? Age and gender differences in the link between marital quality and cardiovascular risks among older adults. Journal of Health and Social Behavior, 55(4), 403–423. 10.1177/0022146514556893 25413802 PMC4325990

[bibr32-08982643221131926] Liu H. Zhang Y. Burgard S. A. Needham B. L. (2019). Marital status and cognitive impairment in the United States: Evidence from the national health and aging trends study. Annals of Epidemiology, 38, 28–34.e22 10.1016/j.annepidem.2019.08.007 31591027 PMC6812624

[bibr33-08982643221131926] Livingston G. Huntley J. Sommerlad A. Ames D. Ballard C. Banerjee S. Brayne C. Burns A. Cohen-Mansfield J. Costafreda S. G. Dias A. Fox N. Gitlin L. N. Howard R. Kales H. C. Kivimaki M. Larson E. B. Ogunniyi A. Cooper C. (2020). Dementia prevention, intervention, and care: 2020 report of the Lancet commission. The Lancet, 396(10248), 413–446. 10.1016/s0140-6736(20)30367-6 PMC7392084 32738937

[bibr34-08982643221131926] Luo M. Macdonald B. Hülür G. (2022). Not “the more the merrier”: Diminishing returns to daily face-to-face social interaction frequency for well-being in older age. The Journals of Gerontology: Series B, 77(8), 1431–1441. 10.1093/geronb/gbac010 35077534

[bibr35-08982643221131926] Martikainen P. Martelin T. Nihtilä E. Majamaa K. Koskinen S. (2005). Differences in mortality by marital status in Finland from 1976 to 2000: Analyses of changes in marital-status distributions, socio-demographic and household composition, and cause of death. Population Studies, 59(1), 99–115. 10.1080/0032472052000332737 15764137

[bibr36-08982643221131926] Mastekaasa A. (1992). Marriage and psychological well-being: Some evidence on selection into marriage. Journal of Marriage and the Family, 54(4), 901–911. 10.2307/353171

[bibr37-08982643221131926] Mata J. Richter D. Schneider T. Hertwig R. (2018). How cohabitation, marriage, separation, and divorce influence BMI: A prospective panel study. Health Psychology, 37(10), 948–958. 10.1037/hea0000654 30234354

[bibr38-08982643221131926] Moms J. Heyman A. Mohs R. Hughes J. van Belle G. Fillenbaum G. Mellits E. D. Clark C. (1989). The Consortium to establish a registry for Alzheimer’s disease (CERAD). Part I. Clinical and neuropsychological assesment of Alzheimer’s disease. Neurology, 39(9), 1159–1159. 10.1212/wnl.39.9.1159 2771064

[bibr39-08982643221131926] Murray J. E. (2000). Marital protection and marital selection: Evidence from a historical-prospective sample of American men. Demography, 37(4), 511–521. 10.1353/dem.2000.0010 11086576

[bibr40-08982643221131926] Najar J. Aakre J. A. Vassilaki M. Wetterberg H. Rydén L. Zettergren A. Skoog I. Jack C. R. Knopman D. S. Kern S. Mielke M. M. Petersen R. C. (2021). Sex difference in the relation between marital status and dementia risk in two population-based cohorts. Journal of Alzheimer's Disease, 83(3), 1269–1279. 10.3233/jad-210246 PMC8490335 34420952

[bibr41-08982643221131926] Nasreddine Z. S. Phillips N. A. Bédirian V. Charbonneau S. Whitehead V. Collin I. Cummings J. L. Chertkow H. (2005). The montreal cognitive assessment, MoCA: A brief screening tool for mild cognitive impairment. Journal of the American Geriatrics Society, 53(4), 695–699. 10.1111/j.1532-5415.2005.53221.x 15817019

[bibr42-08982643221131926] Næss Ø. Søgaard A. J. Arnesen E. Beckstrøm A. C. Bjertness E. Engeland A. Hjort P. F. Holmen J. Magnus P. Njolstad I. Tell G. S. Vatten L. Vollset S. E. Aamodt G. Njølstad I. (2008). Cohort profile: Cohort of Norway (CONOR). International Journal of Epidemiology, 37(3), 481–485. 10.1093/ije/dym217 17984119 PMC2409050

[bibr43-08982643221131926] Nguyen T. T. Tchetgen E. J. T. Kawachi I. Gilman S. E. Walter S. Liu S. Y. Manly J. J. Glymour M. M. (2016). Instrumental variable approaches to identifying the causal effect of educational attainment on dementia risk. Annals of Epidemiology, 26(1), 71–76.e73 10.1016/j.annepidem.2015.10.006 26633592 PMC4688127

[bibr44-08982643221131926] Pesando L. M. Castro A. F. Andriano L. Behrman J. A. Billari F. Monden C. Furstenberg F. Kohler H.-P. (2018). Global family change: Persistent diversity with development. 10.1111/padr.12209 PMC8301234 34305197

[bibr45-08982643221131926] Pinto T. C. Machado L. Bulgacov T. M. Rodrigues-Júnior A. L. Costa M. L. Ximenes R. C. Sougey E. B. (2019). Is the montreal cognitive assessment (MoCA) screening superior to the mini-mental state examination (MMSE) in the detection of mild cognitive impairment (MCI) and Alzheimer’s disease (AD) in the elderly? International Psychogeriatrics, 31(4), 491–504. 10.1017/s1041610218001370 30426911

[bibr46-08982643221131926] Portacolone E. Rubinstein R. L. Covinsky K. E. Halpern J. Johnson J. K. (2019). The precarity of older adults living alone with cognitive impairment. The Gerontologist, 59(2), 271–280. 10.1093/geront/gnx193 29373676 PMC6417768

[bibr48-08982643221131926] Ruano L. Araújo N. Branco M. Barreto R. Moreira S. Pais R. Cruz V. T. Lunet N. Barros H. (2019). Prevalence and causes of cognitive impairment and dementia in a population-based cohort from Northern Portugal. American Journal of Alzheimer's Disease & Other Dementias®, 34(1), 49–56. 10.1177/1533317518813550 PMC10852416 30514090

[bibr49-08982643221131926] Sachdev P. S. Lipnicki D. M. Crawford J. Reppermund S. Kochan N. A. Trollor J. N. Wen W. Draper B. Slavin M. J. Lux O. Mather K. A. Brodaty H. Team A. S. Kang K. (2013). Factors predicting reversion from mild cognitive impairment to normal cognitive functioning: A population-based study. Plos One, 8(3), Article e59649. 10.1371/journal.pone.0059649 23544083 PMC3609866

[bibr50-08982643221131926] Schmitt F. Saxton J. Ferris S. Mackell J. Sun Y. (2013). Evaluation of an 8-item Severe Impairment Battery (SIB-8) vs. the full SIB in moderate to severe Alzheimer's disease patients participating in a donepezil study. International Journal of Clinical Practice, 67(10), 1050–1056. 10.1111/ijcp.12188 24073978 PMC3930878

[bibr51-08982643221131926] Seaman S. R. White I. R. Copas A. J. Li L. (2012). Combining multiple imputation and inverse-probability weighting. Biometrics, 68(1), 129–137. 10.1111/j.1541-0420.2011.01666.x 22050039 PMC3412287

[bibr53-08982643221131926] Shiri-Feshki M. (2009). Rate of progression of mild cognitive impairment to dementia-Meta-analysis of 41 robust inception cohort studies. 10.1111/j.1600-0447.2008.01326.x 19236314

[bibr54-08982643221131926] Skirbekk V. Hardy M. Strand B. H. (2018). Women’s spousal choices and a man’s handshake: Evidence from a Norwegian study of cohort differences. SSM-population Health, 5, 1–7. 10.1016/j.ssmph.2018.04.004 30073184 PMC6069588

[bibr55-08982643221131926] Smock P. J. Schwartz C. R. (2020). The demography of families: A review of patterns and change. Journal of Marriage and Family, 82(1), 9–34. 10.1111/jomf.12612 32612304 PMC7329188

[bibr56-08982643221131926] Sommerlad A. Ruegger J. Singh-Manoux A. Lewis G. Livingston G. (2018). Marriage and risk of dementia: Systematic review and meta-analysis of observational studies. Journal of Neurology, Neurosurgery & Psychiatry, 89(3), 231–238. 10.1136/jnnp-2017-316274 29183957 PMC5869449

[bibr57-08982643221131926] SSB (2021). Divorce rates by age and sex. https://www.ssb.no/statbank/table/05702/

[bibr58-08982643221131926] Statistics Norway (2021). Population statistics. www.ssb.no/befolkning

[bibr59-08982643221131926] Statistics Norway (2022). Divorce rates by ages in Norway. https://www.ssb.no/en/statbank/table/05702

[bibr60-08982643221131926] Statacorp (2022). Stata SE v 17. stata.com

[bibr61-08982643221131926] Stern Y. (2012). Cognitive reserve in ageing and Alzheimer's disease. The Lancet Neurology, 11(11), 1006–1012. 10.1016/s1474-4422(12)70191-6 23079557 PMC3507991

[bibr62-08982643221131926] Sundström A. Westerlund O. Kotyrlo E. (2016). Marital status and risk of dementia: A nationwide population-based prospective study from Sweden. BMJ open, 6(1), Article e008565. 10.1136/bmjopen-2015-008565 PMC4716184 26729377

[bibr63-08982643221131926] Tangen G. G. Langballe E. M. Strand B. H. (2020). Subjective memory impairment, instrumental activities of daily living and longitudinal effect on mortality among older adults in a population-based cohort study: The HUNT Study. Scandinavian Journal of Public Health, 48(8), 825–831. 10.1177/1403494819885234 31825300

[bibr64-08982643221131926] Tosi M. van den Broek T. (2020). Gray divorce and mental health in the United Kingdom. Social Science & Medicine, 256, 113030. 10.1016/j.socscimed.2020.113030 32450471

[bibr65-08982643221131926] Tumin D. Zheng H. (2018). Do the health benefits of marriage depend on the likelihood of marriage? Journal of Marriage and Family, 80(3), 622–636. 10.1111/jomf.12471

[bibr66-08982643221131926] Umberson D. Thomeer M. B. (2020). Family matters: Research on family ties and health, 2010 to 2020. Journal of Marriage and Family, 82(1), 404–419. 10.1111/jomf.12640 33867573 PMC8048175

[bibr67-08982643221131926] VanderWeele T. (2015). Explanation in causal inference: Methods for mediation and interaction. Oxford University Press.

[bibr68-08982643221131926] Vikat A. Jones C. (2014). Indicators of gender equality. United Nations Economic.

[bibr69-08982643221131926] Wadsworth T. (2016). Marriage and subjective well-being: How and why context matters. Social Indicators Research, 126(3), 1025–1048. 10.1007/s11205-015-0930-9

[bibr70-08982643221131926] Waite L. Gallagher M. (2002). The case for marriage: Why married people are happier, healthier and better off financially. Broadway Books.

[bibr71-08982643221131926] Wilson C. M. Oswald A. J. (2005). How does marriage affect physical and psychological health? A survey of the longitudinal evidence. A Survey of the Longitudinal Evidence.

[bibr72-08982643221131926] World Health Organization . (2021). Dementia. https://www.who.int/news-room/fact-sheets/detail/dementia

[bibr73-08982643221131926] Zahl-Olsen R. Thuen F. Espehaug B. (2019). Divorce and remarriage in Norway: A prospective cohort study between 1981 and 2013. Journal of Divorce & Remarriage, 60(8), 600–611. 10.1080/10502556.2019.1619378

