# Supplementary material for: Marital Histories and Associations With Later-Life Dementia and Mild Cognitive Impairment Risk in the HUNT4 70+ Study in Norway
Source: J Aging Health. 2022 Nov 2;35(7-8):543–55. doi: 10.1177/08982643221131926 (PMC10151439; doi:10.1177/08982643221131926)
Supplement: Supplemental Material - Marital Histories and Associations With Later-Life Dementia and Mild Cognitive Impairment Risk in the HUNT4 70+ Study in Norway [file sj-pdf-1-jah-10.1177_08982643221131926.pdf]

Supplementary Materials

Weights

Table S1 displays the weights used in our analyses to correct for non-participation bias due to age, sex, and education. We used a logistic regression analysis to estimate the probability of participation in HUNT4 70+ based on age, sex, and education. Data on age, sex and educational level were provided by Statistics Norway (Statistics Norway, 2021). The inverse of the probability of participation constituted the weights. Tertiary-educated women in the younger age groups had the highest probability of participation and were thereby assigned the lowest weight (1.44), while older men with primary education were underrepresented and hence assigned the highest weight (2.89).

Table S1

Inverse probability weights, HUNT4 70+

		Weight	
Education	Age	Men	Women
Primary	70-74	2.52	2.36
	75-79	2.48	2.32
	80-84	2.73	2.54
	85-89	2.89	2.69
	90+	2.64	2.46
Secondary	70-74	1.84	1.75
	75-79	1.82	1.73
	80-84	1.96	1.86
	85-89	2.05	1.94
	90+	1.91	1.81
Tertiary	70-74	1.51	1.46
	75-79	1.50	1.44
	80-84	1.58	1.52
	85-89	1.63	1.57
	90+	1.55	1.49

Table S2

Results of the multinomial logistic regression (relative risk ratios and 95% confidence intervals) for MCI or dementia diagnosis versus no cognitive impairment by marital history. Model covariates in parentheses.

	N (%)	Model 1 (age, sex)		Model 2 (+education)		Model 3 (+number of children)		Model 4 (+physical health risk factors[a])		Model 5 (+ mental distress, no close friends)	
		MCI	Dementia	MCI	Dementia	MCI	Dementia	MCI	Dementia	MCI	Dementia
Continuously married	6141 (70.5)	Reference									
Intermittently married	985 (11.3)	**1.16 (1.00, 1.34)***	1.21 (0.97, 1.51)	1.16 (0.99, 1.34)	1.19 (0.95, 1.50)	1.14 (0.91, 1.44)	1.14 (0.91, 1.44)	1.14 (0.98, 1.33)	1.14 (0.91, 1.43)	1.13 (0.97, 1.32)	1.11 (0.88, 1.40)
Intermittently. divorced	461 (5.3)	0.95 (0.77, 1.18)	**1.49 (1.09, 2.04)***	0.96 (0.77, 1.19)	**1.50 (1.09, 2.06)***	0.95 (0.77, 1.19)	**1.45 (1.05, 2.00)***	0.95 (0.76, 1.18)	**1.46 (1.05, 2.02)***	0.93 (0.75, 1.16)	1.37 (0.98, 1.90)
Unmarried	404 (4.6)	**1.48 (1.19, 1.85)***	**1.77 (1.27, 2.47)***	1.43 (1.15, 1.78)*	**1.73 (1.24, 2.40)***	1.32 (0.98, 1.77)	1.17 (0.77, 1.78)	1.32 (0.98, 1.78)	1.19 (0.78, 1.81)	1.32 (0.98, 1.78)	1.18 (0.78, 1.80)
Widowed	378 (4.3)	1.16 (0.92, 1.34)	1.13 (0.80, 1.59)	1.12 (0.88, 1.42)	1.04 (0.73, 1.48)	1.11 (0.88, 1.41)	1.04 (0.73, 1.48)	1.11 (0.87, 1.40)	1.03 (0.72, 1.47)	1.10 (0.87, 1.40)	1.02 (0.72, 1.46)
Continuously divorced	337 (3.9)	1.20 (0.94, 1.54)	**1.84 (1.26, 2.68)***	1.13 (0.88, 1.44)	**1.66 (1.14, 2.43)***	1.11 (0.87, 1.42)	**1.60 (1.10, 2.34)***	1.10 (0.86, 1.41)	**1.60 (1.09, 2.34)***	1.10 (0.86, 1.41)	**1.60 (1.09, 2.34)***

Notes. Analyses based on *N*=8706 participants aged 70 to 87 in HUNT4 70+. Analyses were weighted for non-participation using inverse probability weights; missing values were imputed with multiple imputation (chained logit, 30 samples). [a]Smoking, hypertension, obesity, physically inactive, diabetes. *$p < .05$

Figure S1

Lexis diagram showing register-based marital histories from age 44 to 68 years for each birth cohort born 1931-1949 (green). Clinical MCI/dementia diagnosis was assessed in HUNT4 70+ at age 70-88 years in 2017-19 for these birth cohorts (red)

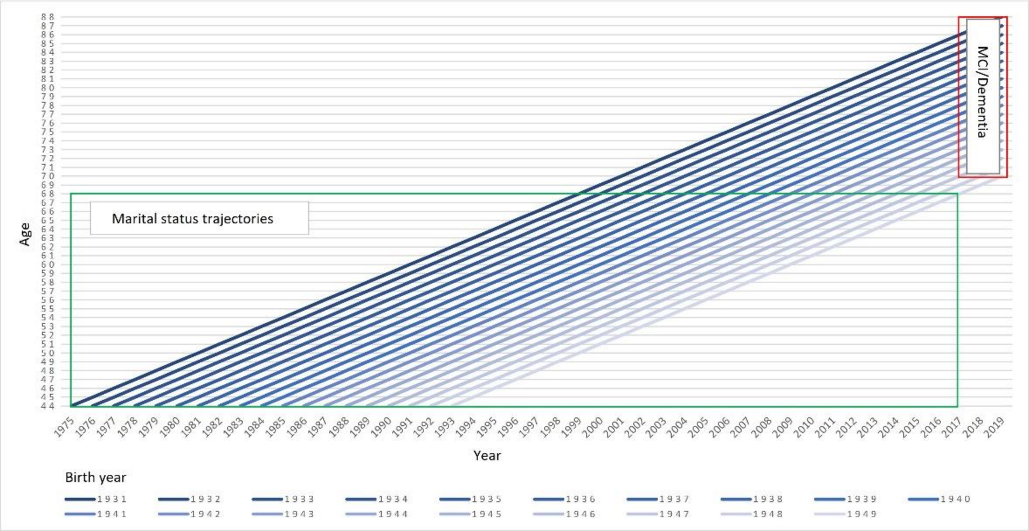

Figure S2

Results of the sex-specific multinomial logistic regression analyses (relative risk ratios, RRR and 95% confidence intervals) for MCI or dementia diagnosis by marital history with continuously married as the reference group. Analyses based on *N*=8706 participants aged 70 to 87 in HUNT4 70+. Analyses were weighted for non-response using inverse probability weights; missing values were imputed with multiple imputation (chained logit, 30 samples).

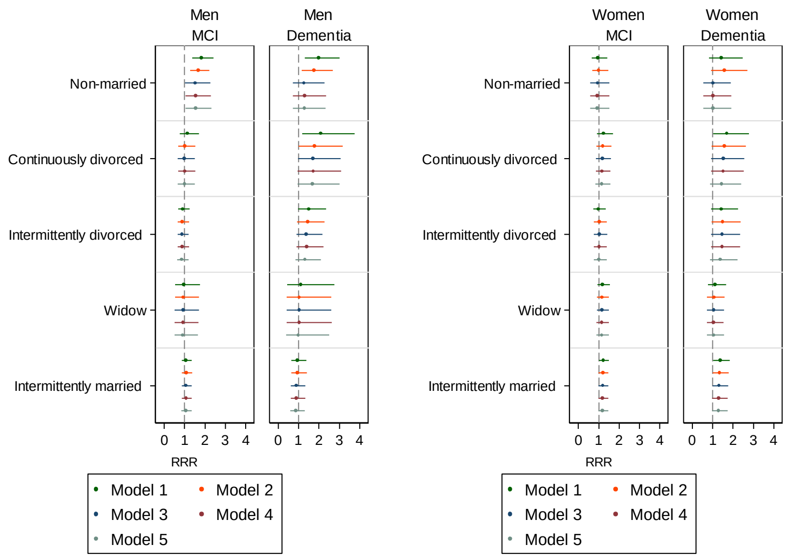

Model 1 was adjusted for age; Model 2 was additionally adjusted for education; Model 3 was additionally adjusted for number of children; Model 4 was additionally adjusted for smoking, hypertension, obesity, physical inactivity, and diabetes; Model 5 was additionally adjusted for mental distress and no close friends.
